# Supplementary material for: Hepatic metabolism of grazing cows of two Holstein strains under two feeding strategies with different levels of pasture inclusion
Source: PLoS One. 2023 Oct 26;18(10):e0290551. doi: 10.1371/journal.pone.0290551 (PMC10602316; doi:10.1371/journal.pone.0290551)

Gel 1

NZH NAH

FixP MaxP FixP MaxP

PM 21 180 21 180 21 180 21 180 Acetyl-BSA

AcK

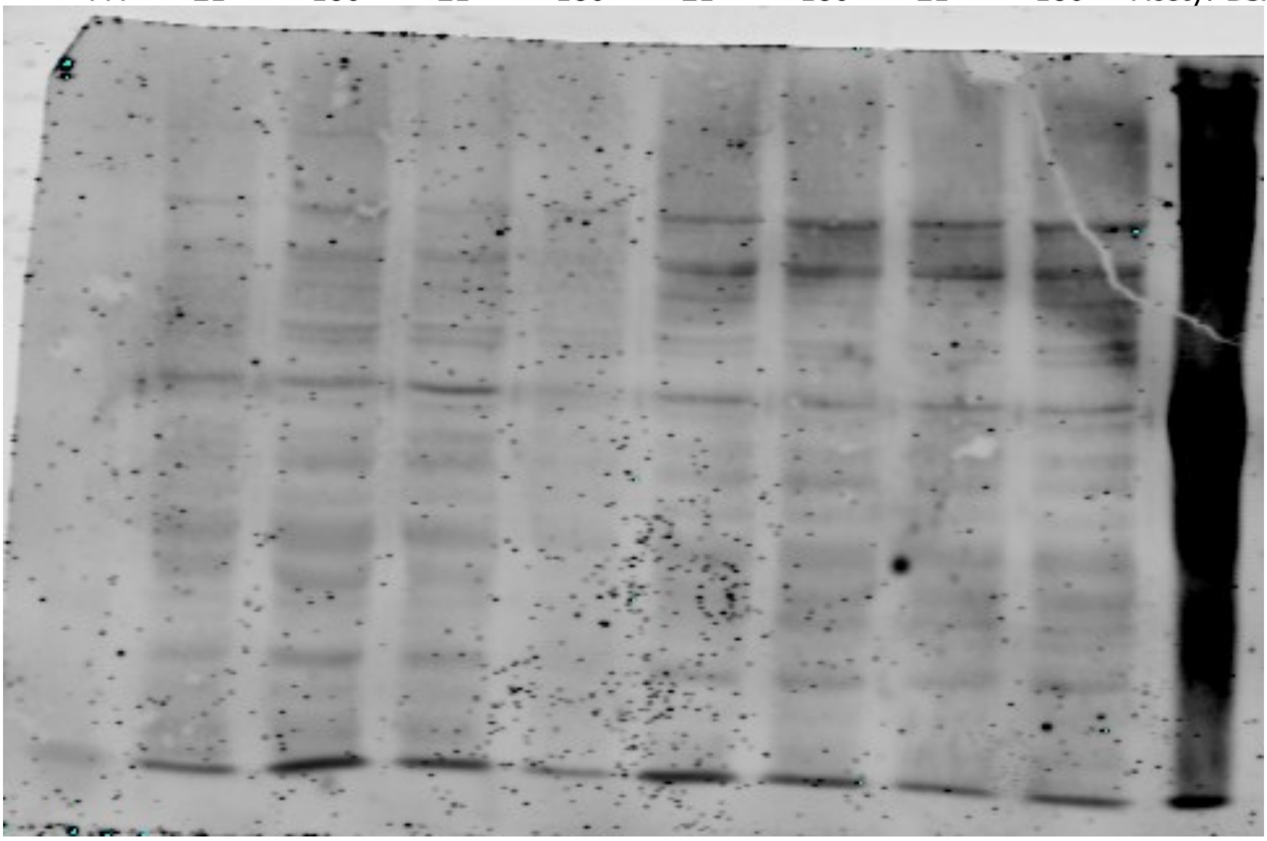

Ponceau

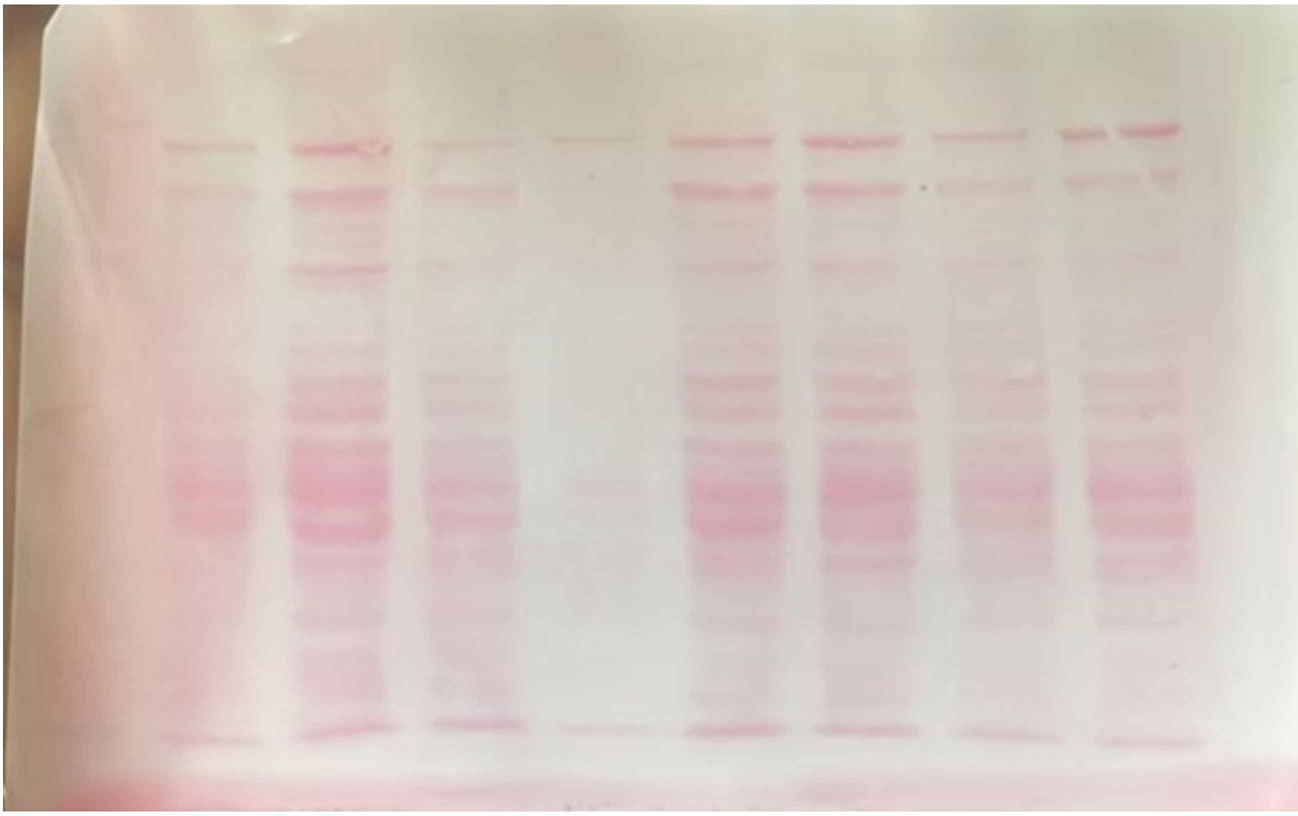

Gel 2

NZH | NAH
FixP | MaxP | FixP | MaxP

PM | 21 | 180 | 21 | 180 | 21 | 180 | 21 | 180 | Acetyl-BSA

AcK

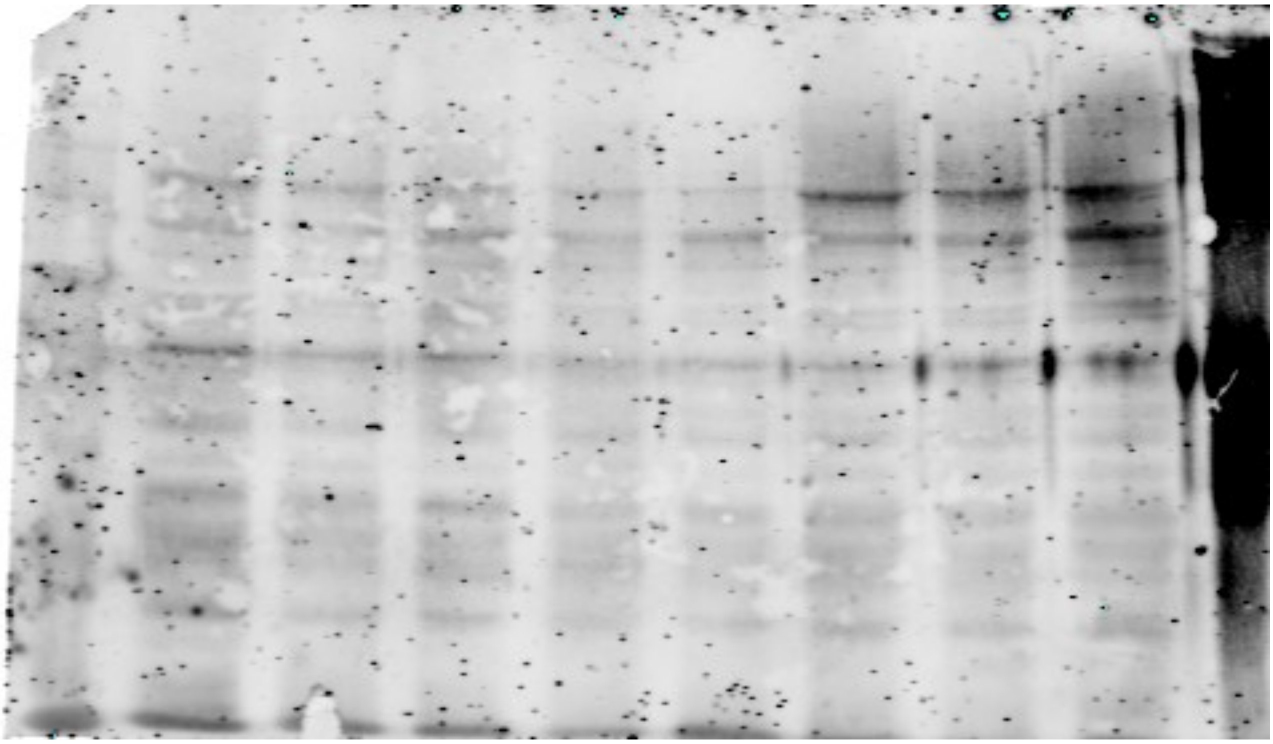

Ponceau

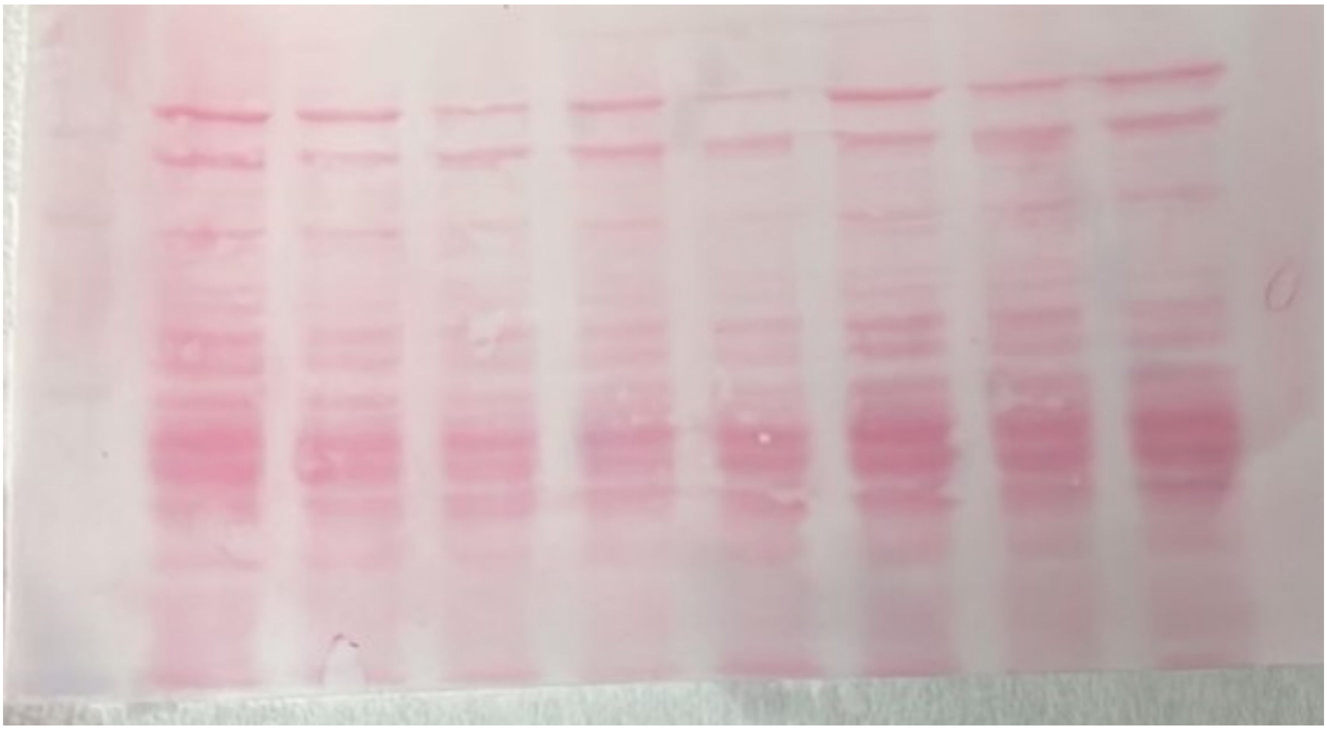

Gel 3

	NZH				NAH				
	FixP		MaxP		FixP		MaxP		
PM	21	180	21	180	21	180	21	180	Acetyl-BSA

AcK

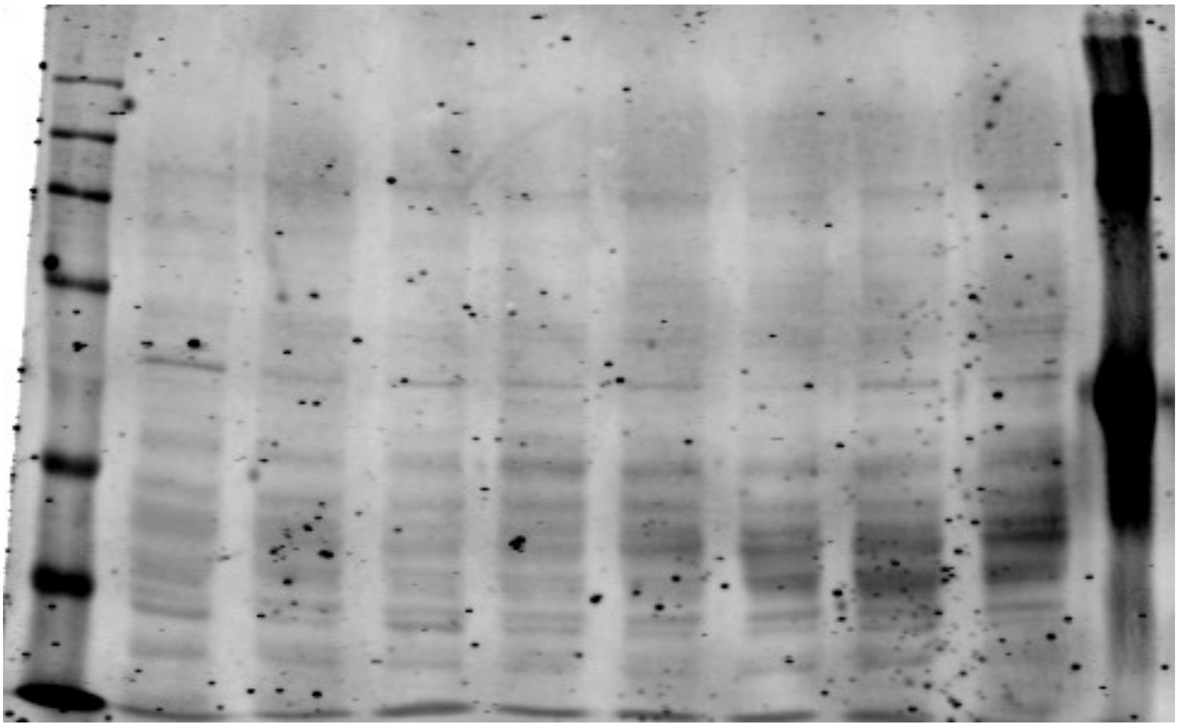

Ponceau

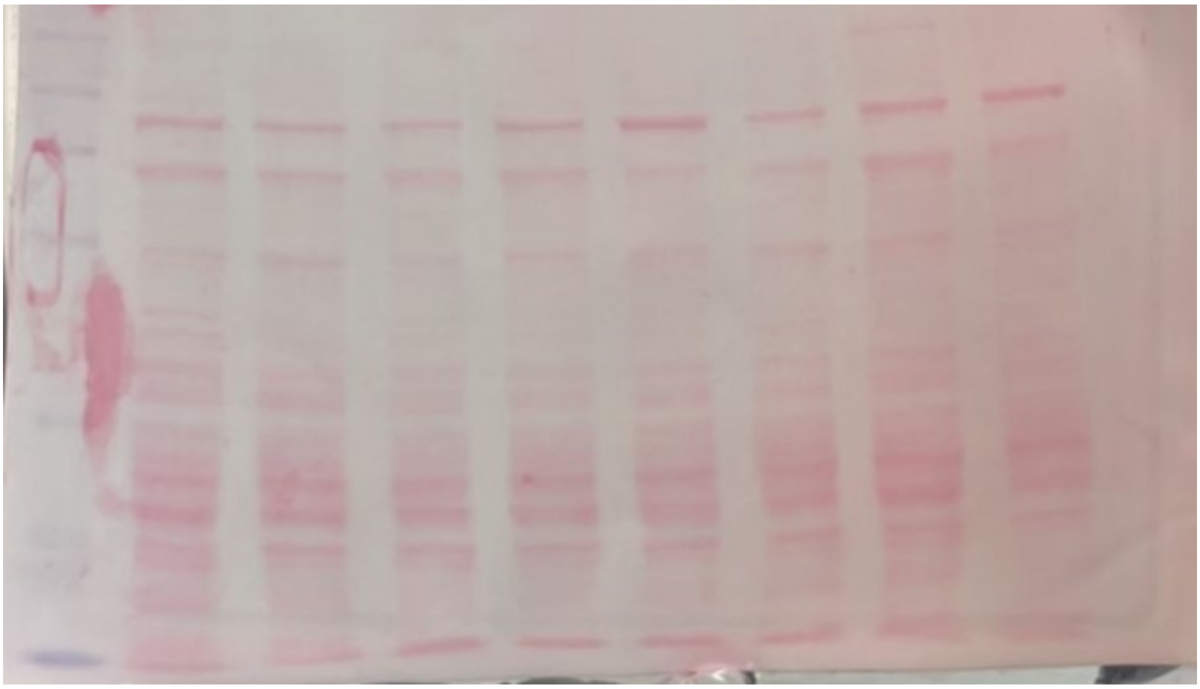

Gel 4

NZH | NAH

FixP | MaxP | FixP | MaxP

PM | 21 | 180 | 21 | 180 | 21 | 180 | 21 | 180 | Acetyl-BSA

AcK

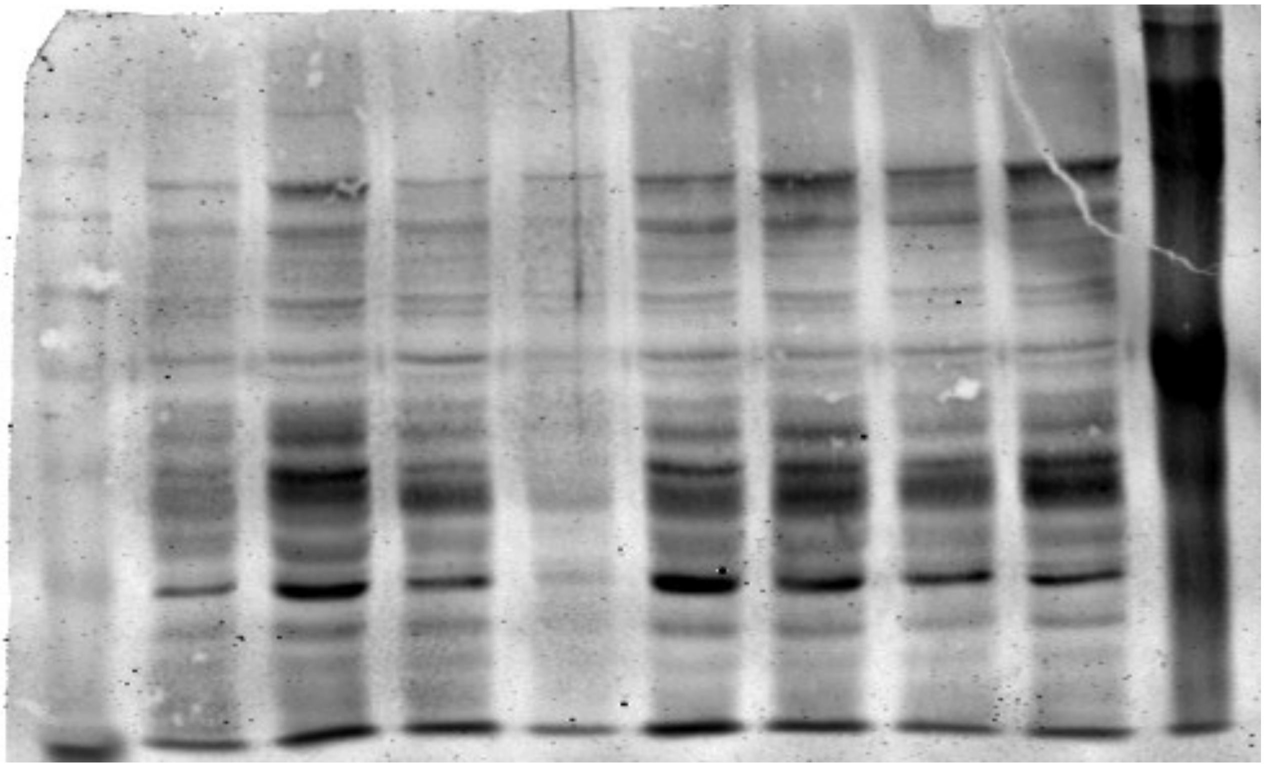

Ponceau

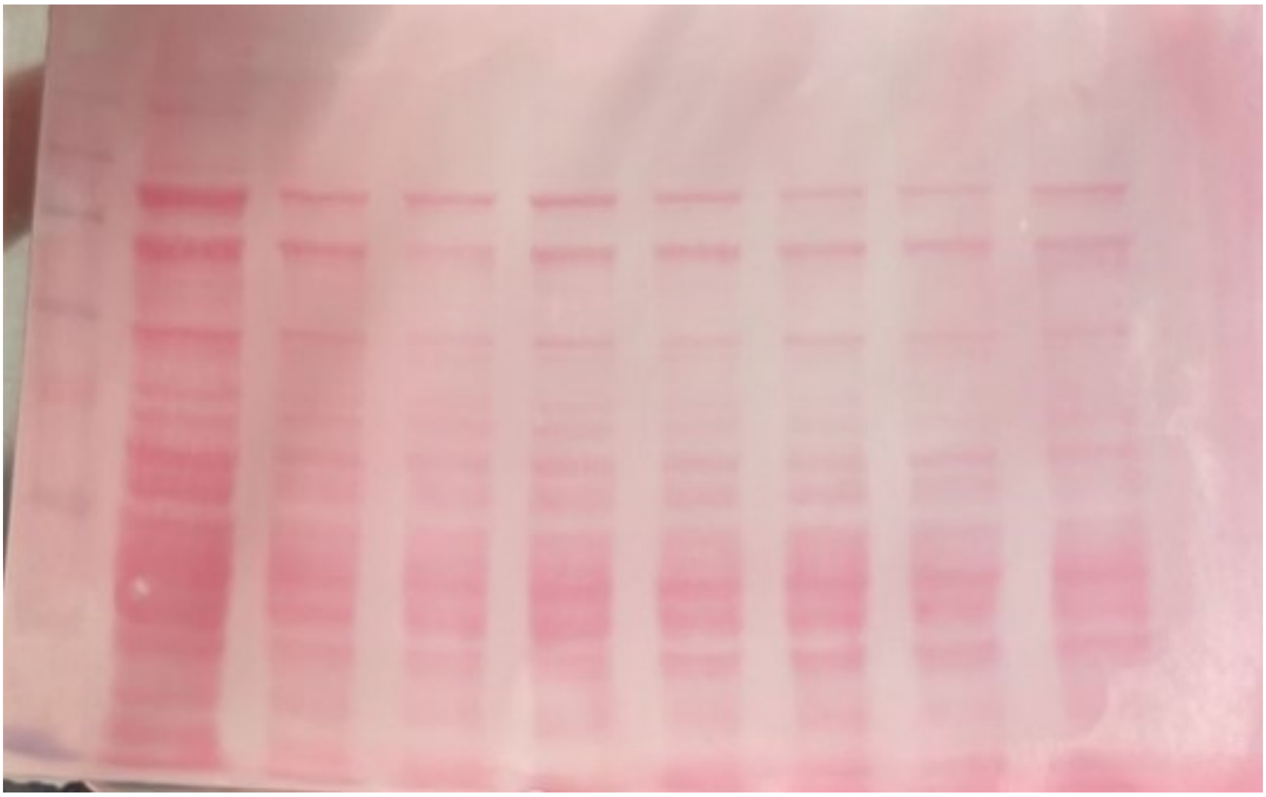

Gel 5

	NZH				NAH				
	FixP		MaxP		FixP		MaxP		
PM	21	180	21	180	21	180	21	180	Acetyl-BSA

AcK

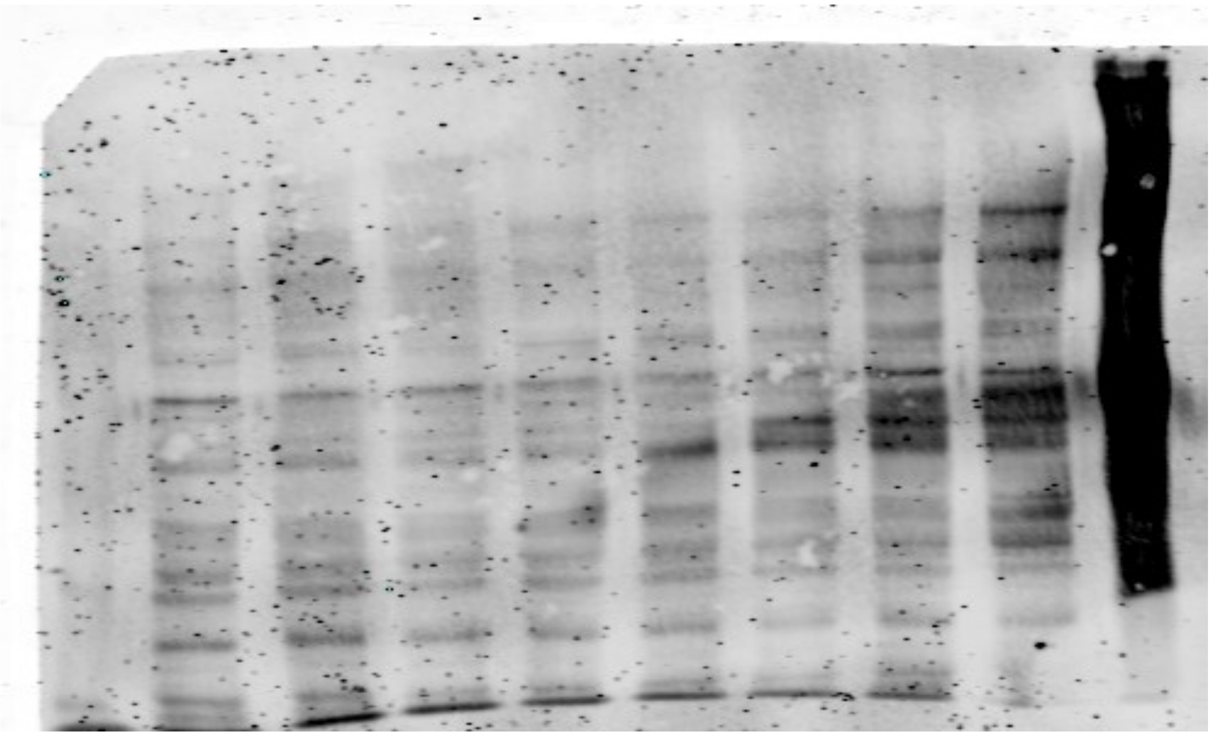

Ponceau

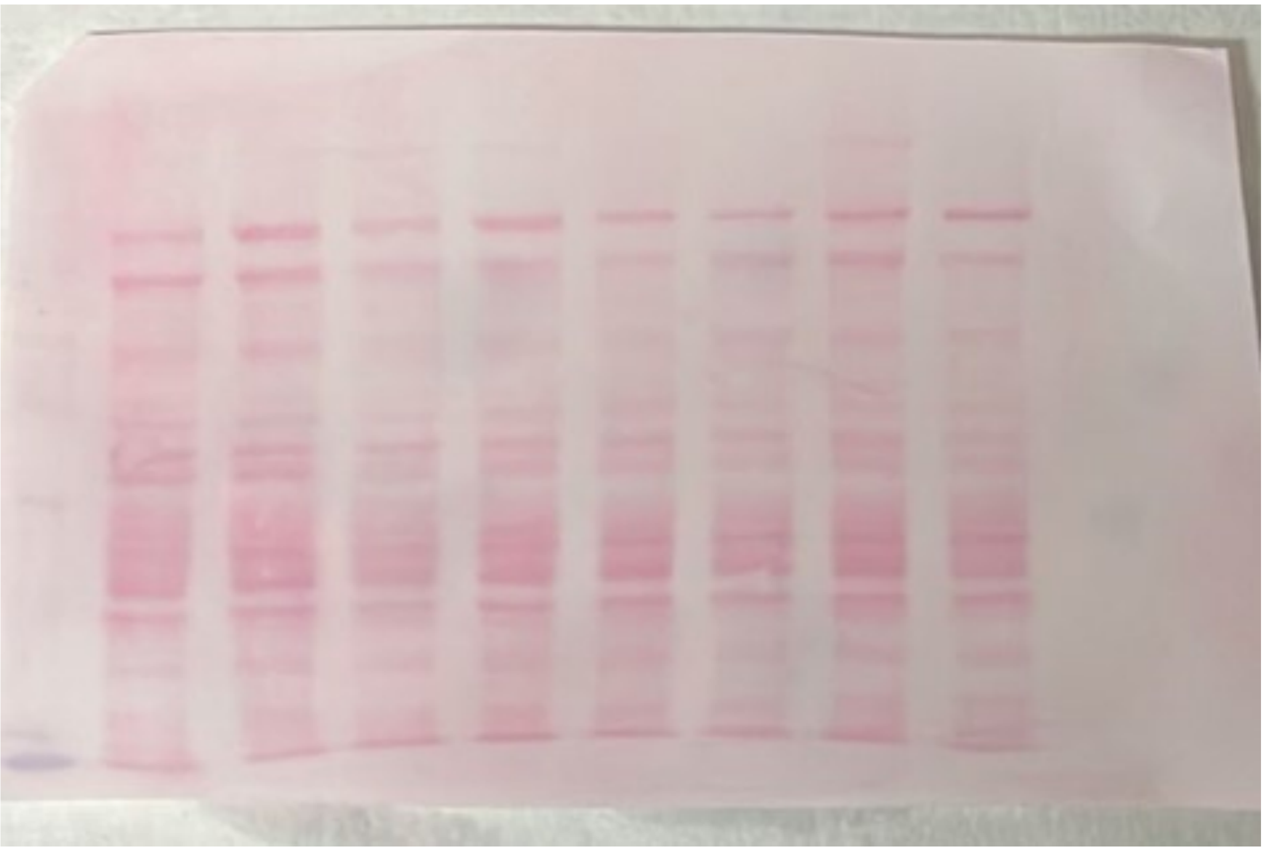

Gel 6

NZH | NAH

FixP | MaxP | FixP | MaxP

PM 21 180 21 180 21 180 21 180 Acetyl-BSA

AcK

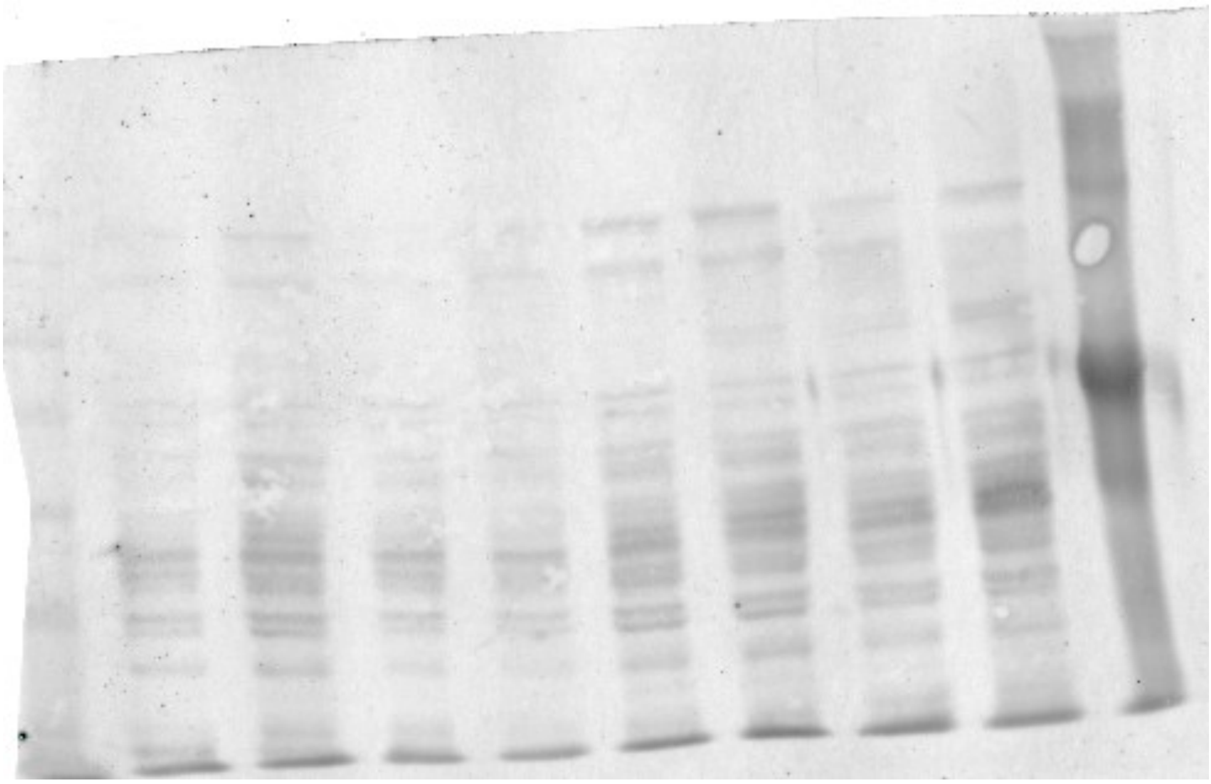

Ponceau

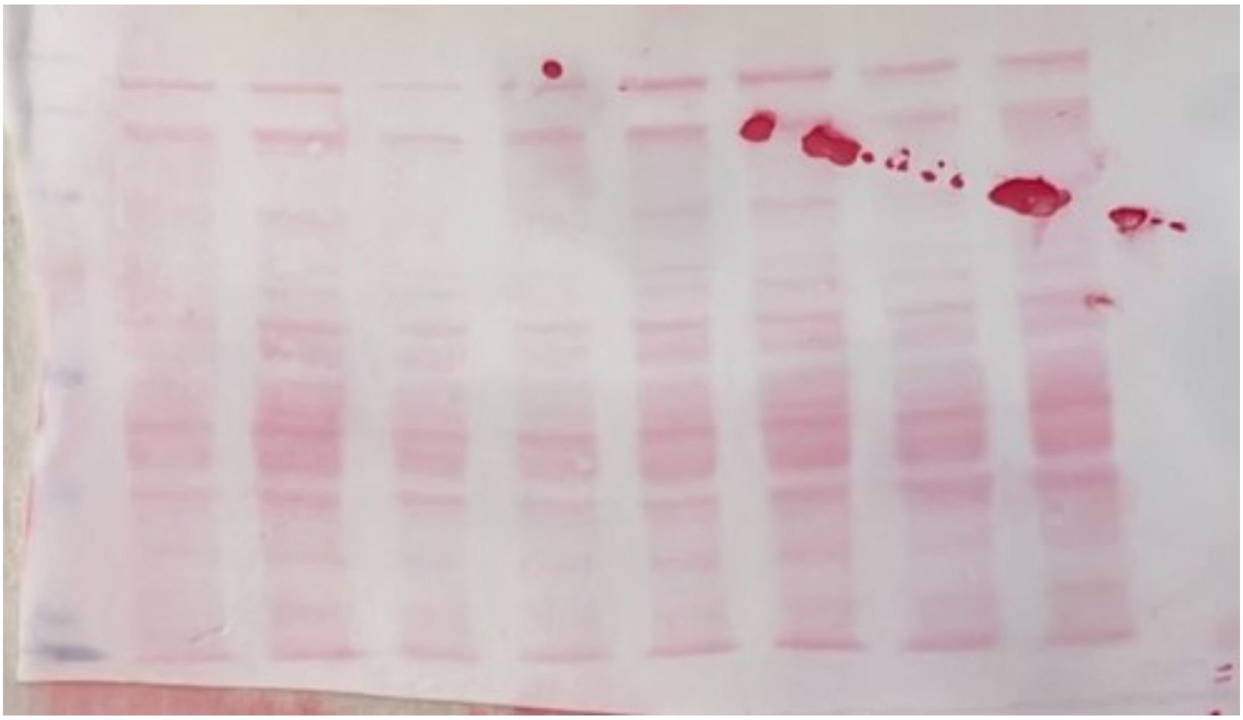

Gel 7

NZH | NAH

FixP | MaxP | FixP | MaxP

PM | 21 | 180 | 21 | 180 | 21 | 180 | 21 | 180 | Acetyl-BSA

AcK

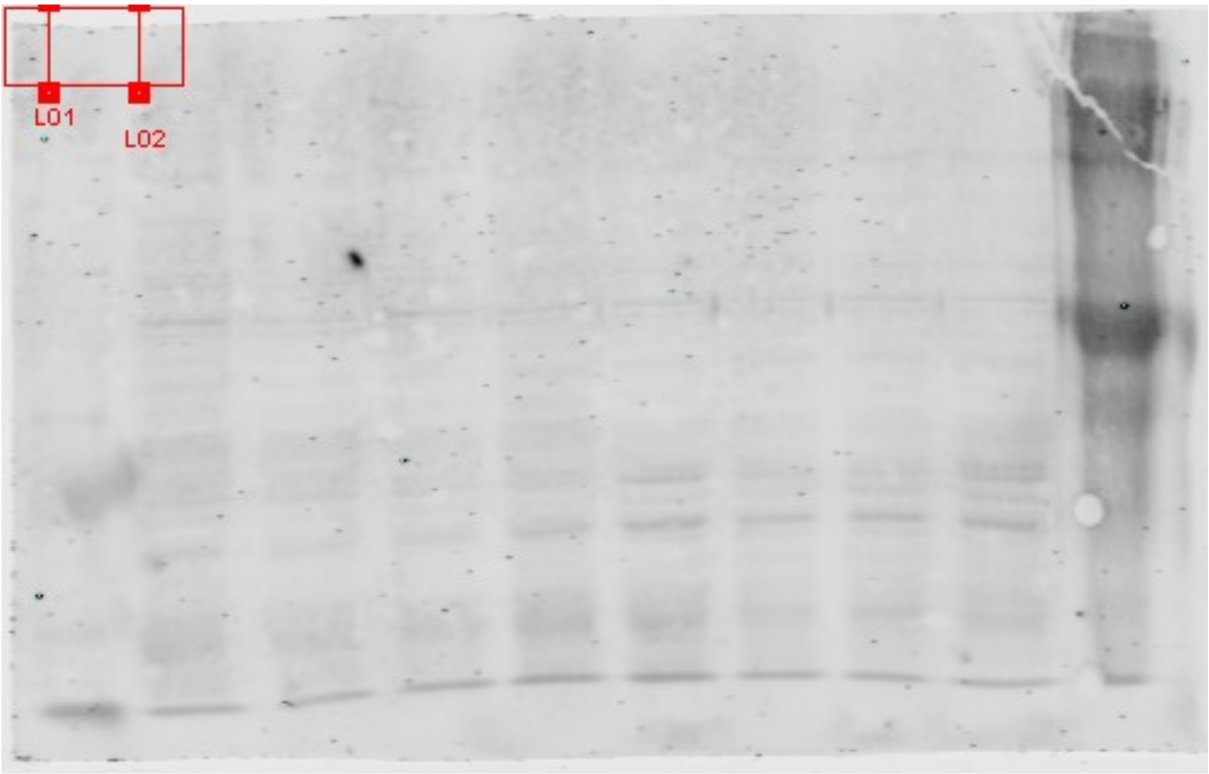

Ponceau

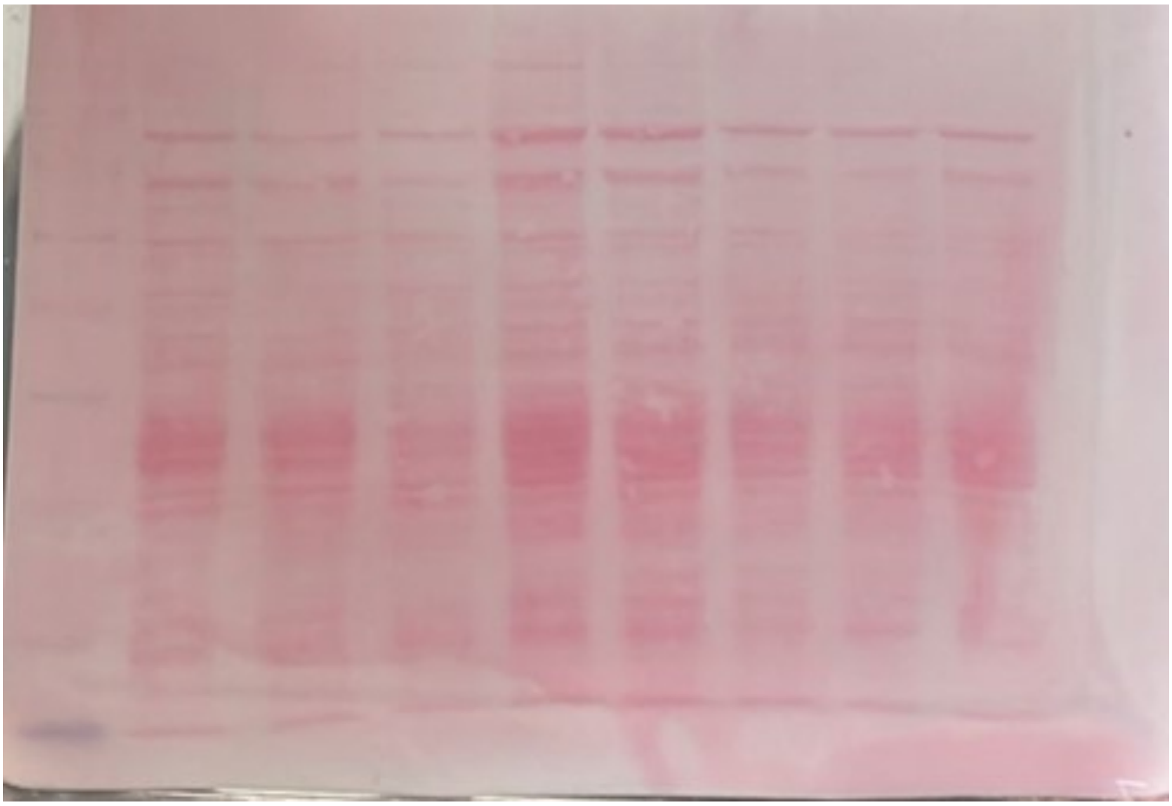

Gel 8

NZH — FixP: 21, 180; MaxP: 21, 180
NAH — FixP: 21, 180; MaxP: 21, 180

PM | 21 | 180 | 21 | 180 | 21 | 180 | 21 | 180 | Acetyl-BSA

AcK

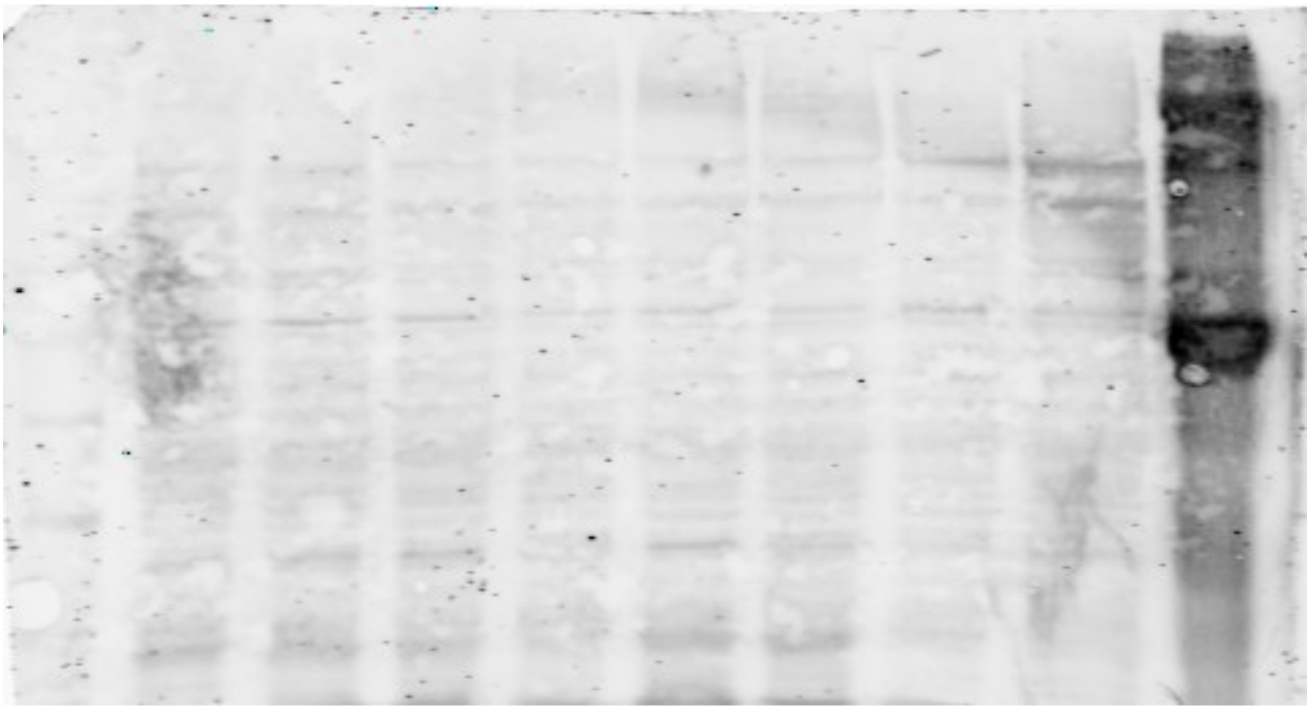

Ponceau

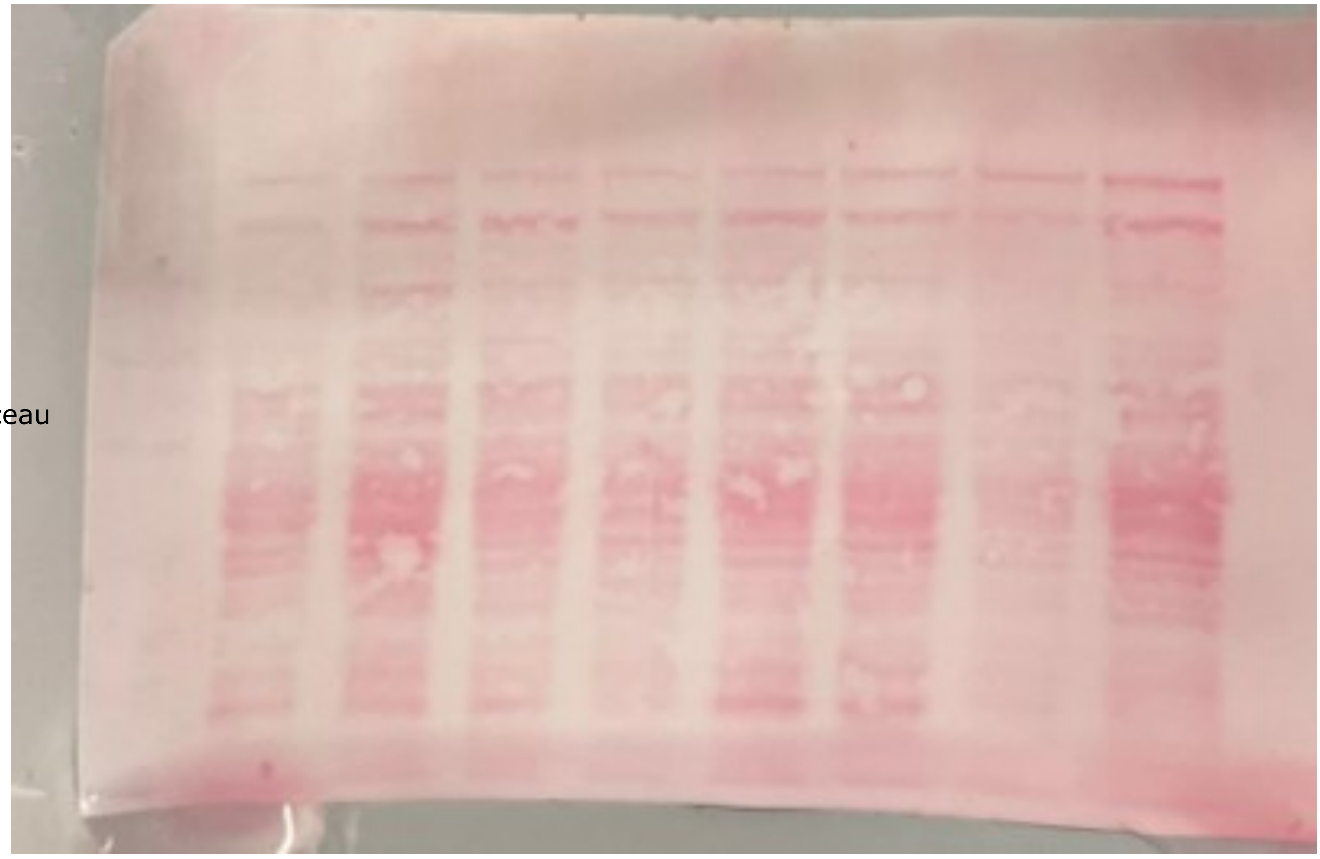

Supplement: S1 File — (N = 8). The last lane is acetylated BSA, bottom panel shows ponceau S staining for protein normalization. Western blots were quantified by densitometry, normalized with the loading control and expressed in relation to the average value of the NZH-FixP treatment at 21 DPP. (PDF) [file pone.0290551.s001.pdf]
