# Supplementary material for: Hepatic metabolism of grazing cows of two Holstein strains under two feeding strategies with different levels of pasture inclusion
Source: PLoS One. 2023 Oct 26;18(10):e0290551. doi: 10.1371/journal.pone.0290551 (PMC10602316; doi:10.1371/journal.pone.0290551)
Supplement: S1 Table — 1Genes: Very long-chain acyl-CoA dehydrogenase (ACADVL), acetyl-CoA acetyltransferase 1 (ACAT1), ß-actin (ACTB), acyl-CoA oxidase 2 (ACOX2), apolipoprotein A4 (APOA4), apolipoprotein A5 (APOA5), apolipoprotein C2 (APOC2), CD36 molecule (CD36), CD40 molecule (CD40), carnitine palmitoyl-transferase 1 (CPT1A), liver fatty acid binding protein (FABP1), fibroblast growth factor 21 (FGF21), hydroxymethylglutaryl-CoA synthase 2 (HMGCS2), hypoxanthine phosphoribosyl transferase (HPRT1); nuclear receptor subfamily 1 group H member 3 (LXRA), nuclear factor kappa B subunit 1 (NFKB1), nuclear factor kappa B inhibitor alpha (NFKB1A), peroxisome proliferator-activated receptor alpha (PPARA), peroxisome proliferator-activated receptor gamma coactivator 1-alpha (PPARGC1A), retinoic acid receptor alpha (RARA), retinoic X receptor alpha (RXRA), retinoic X receptor beta (RXRB), retinoic X receptor gamma (RXRG), sterol regulatory element binding transcription factor 1 (SREBP-1), tumor necrosis factor alpha (TNFA), tumor necrosis factor receptor superfamily member 1A (TNFRSF1A). (DOCX) [file pone.0290551.s002.docx]

| **Gene^1,2^** | | **Accession#^3^** |  | **Primer sequence^4^** | **Length(bp)** | **Efficiency** |
| --- | --- | --- | --- | --- | --- | --- |
| *ACADVL* | | NM_174494.2 | F | CCAGCCCCTGTGGAAAATACTA | 62 | 0.72 |
|  | | | R | GCCCCCGTTACTGATCCAA |  |  |
| *ACAT1* | | NM_001046075.1 | F | AGAGCATGTCCAATGTCCCC | 70 | 0.94 |
|  | | | R | TCTTCAAGCTTTACCCCACCA |  |  |
| *ACOX2* | | NM_001102015.2 | F | CCCTACATGGCATCCTGACT | 186 | 1.44 |
|  | | | R | CATAACAGCCGAGTGCTGAA |  |  |
| *APOA4* | NM_001037480.1 | | F | TGAATCCAGGAAGGATCTGG | 249 | 0.89 |
|  | | | R | CTTGGAAGAGGGTGTTGAGC |  |  |
| *APOA5* | NM_001083492.2 | | F | GACGACCTGTGGGAAGACAT | 218 | 1.15 |
|  | | | R | CTGAGGCCTAGGATGACAGC |  |  |
| *APOC2* | NM_001102380.2 | | F | CAGAGTCTGCCACCTCAGTG | 197 | 1.21 |
|  | | | R | GCCTTGGCTGTATCCCAGTA |  |  |
| *ACTB* | | NM_173979 | F | CTCTTCCAGCCTTCCTTCCT | 178 | 1.00 |
|  | |  | R | GGGCAGTGATCTCTTTCTGC |  |  |
| *CD36* | | NM_001278621.1 | F | ATTTGACCCAGCACTTGAGG | 181 | 1.20 |
|  | |  | R | CGGGTCTGATGAAAGTGGTT |  |  |
| *CD40* | | NM_001105611.2 | F | AGGGCTTTTGGATACCGTCT | 210 | 1.20 |
|  | |  | R | AACAGGACTCCCATCGTGAC |  |  |
| *CPT1A* | | NM_001304989 | F | CAAAACCATGTTGTACAGCTTCCA | 140 | 0.77 |
|  | |  | R | GCTTCCTTCATCAGAGGCTTCA |  |  |
| *FABP1* | | NM_175817 | F | GTTCATCATCACCGCTGGCT | 101 | 1.00 |
|  | |  | R | CCACTGCCTTGATCTTCTCCC |  |  |
| *FGF21* | | XM_024979245.1 | F | CGGATCGCTGCACTTTGAC | 76 | 0.97 |
|  | |  | R | CTGGTAGACGTTGTATCCATCTTCA |  |  |
| *HMGCS2* | | NM_001045883 | F | AGAACGTCTGCCCTCTTTCA | 81 | 0.71 |
|  | |  | R | TACAAGGCTGCTGTGTCCAG |  |  |
| *HPRT1* | | NM_001034035 | F | TGGAGAAGGTGTTTATTCCTC | 105 | 1.00 |
|  | |  | R | CACAGAGGGCCACAATGTGA |  |  |
| *LXRA* | | NM_001014861.1 | F | CATCAACCCCATCTTCGAGT | 235 | 1.37 |
|  | |  | R | GCTCACCAGTTTCATCAGCA |  |  |
| *NFKB1* | | NM_001076409.1 | F | CTGGAAGCACGAATGACAGA | 215 | 1.12 |
|  | |  | R | GTGCTGTCTGGAAGGAAAGC |  |  |
| *NFKB1A* | | NM_001045868.1 | F | CTGCACTTAGCCATCATCCA | 230 | 1.17 |
|  | |  | R | TGCTCACAGGCAAGGTGTAG |  |  |
| *PPARA* | | NM_001034036 | F | CGGTGTCCACGCATGTGA | 56 | 1.20 |
|  | |  | R | TCAGCCGAATCGTTCTCCTAAA |  |  |
| *PPARGC1A* | | NM_177945 | F | TGAACCCAGCTGCTGAAGAG | 216 | 1.28 |
|  | |  | R | AGAACCTGCGGTGTCTTCAG |  |  |
| *RARA* | | NM_001014942.4 | F | CAAGACAAATCCTCCGGCTA | 217 | 0.85 |
|  | |  | R | TGTTCCGGTCATTTCTCACA |  |  |
| *RXRA* | | NM_001304343.1 | F | TCCAAAGATGGCTTTCAACC | 150 | 1.45 |
|  | |  | R | AGGAGCTGAAACCAGGACAA |  |  |
| *RXRB* | | NM_001083640.1 | F | GGCAAACACTACGGGGTTTA | 181 | 1.56 |
|  | |  | R | CCTCCCTCTTCATGCCAGTA |  |  |
| *RXRG* | | NM_001075408.1 | F | ATGAAGATATGCCCGTGGAG | 161 | 0.81 |
|  | |  | R | GGCCCATTCAACGAGAGTAA |  |  |
| *SREBP-1* | | NM_001113302.1 | F | CTACATCCGCTTCCTTCAGC | 93 | 1.12 |
|  | |  | R | TCCTTCAGCGATTTGCTTTT |  |  |
| *TNFA* | | NM_174197.2 | F | AACTCTCCCTTCCTGCCAAT | 169 | 1.24 |
|  | |  | R | GGACACCTTGACCTCCTGAA |  |  |
| *TNFRSF1A* | | NM_174674.2 | F | TCCAGTCCTGTCTCCATTCC | 236 | 1.24 |
|  | |  | R | CTGGCTTCCCACTTCTGAAC |  |  |
