# Supplementary material for: Hepatic metabolism of grazing cows of two Holstein strains under two feeding strategies with different levels of pasture inclusion
Source: PLoS One. 2023 Oct 26;18(10):e0290551. doi: 10.1371/journal.pone.0290551 (PMC10602316; doi:10.1371/journal.pone.0290551)
Supplement: S2 Table — Data are shown as least square means ± standard error. ABabMeans having different uppercase superscripts differ significantly within feeding strategy across time period (P < 0.05). Means having different lowercase superscripts differ significantly within strain across time period (P < 0.05), while XYxymeans denote tendencies (0.05 < P < 0.1). N = 10–12. DIM: Days in milk; FS: Feeding strategy. 1Oxygen consumption rate measurements of liver biopsies were obtained after addition of 10 mM glutamate and 5 mM malate, 4 μM ADP, 2 μM oligomycin, up to 4 μM carbonyl cyanide-p- trifluoromethoxyphenylhydrazone and 0.5 μM rotenone, and 2.5 μM antimycin. Oxygen consumption rates are expressed as pmol of O2 per min per mg of wet weight. (DOCX) [file pone.0290551.s003.docx]

| *Complex-I respiratory parameters^1^* | DIM | Treatments | | | | SEM | P-value | | | | | | |
| --- | --- | --- | --- | --- | --- | --- | --- | --- | --- | --- | --- | --- | --- |
|  |  | FixP | | MaxP | |  | DIM | Strain | FS | DIM x Strain | DIM x FS | Strain x FS | DIM x FS x Strain |
|  |  | NZH | NAH | NZH | NAH |  |  |  |  |  |  |  |  |
| State 3 respiration | -45 | 11 | 12 | 19 | 13 | 2 | < 0.05 | 0.32 | 0.95 | 0.32 | 0.18 | 0.19 | 0.75 |
|  | 21 | 12 | 14 | 12 | 12 |  |  |  |  |  |  |  |  |
|  | 180 | 14 | 17 | 15 | 12 |  |  |  |  |  |  |  |  |
| State 4 respiration | -45 | 2.8 | 2.9 | 4.0 | 3.5 | 0.7 | < 0.001 | 0.27 | 0.95 | 0.27 | 0.18 | 0.45 | 0.15 |
|  | 21 | 4.5 | 2.9 | 3.5 | 3.4 |  |  |  |  |  |  |  |  |
|  | 180 | 4.4 | 5.4 | 4.2 | 5.6 |  |  |  |  |  |  |  |  |
| Maximum respiratory capacity | -45 | 11 | 12 | 19 | 13 | 2 | 0.14 | 0.16 | 0.97 | 0.16 | 0.14 | < 0.05 | 0.87 |
|  | 21 | 10 | 14 | 12 | 13 |  |  |  |  |  |  |  |  |
|  | 180 | 14 | 17 | 16 | 12 |  |  |  |  |  |  |  |  |
| Oligomycin-resistant respiration | -45 | 4.0 | 4.3 | 5.0 | 3.8 | 0.6 | < 0.01 | 0.41 | 0.58 | 0.41 | 0.98 | 0.18 | 0.49 |
|  | 21 | 5.8 | 4.9 | 5.9 | 5.5 |  |  |  |  |  |  |  |  |
|  | 180 | 4.9 | 6.3 | 6.2 | 5.6 |  |  |  |  |  |  |  |  |
| Oligomycin-sensitive respiration | -45 | 7^A^ | 7.7^A^ | 13^A^ | 9.2^A^ | 1.5 | < 0.001 | 0.92 | 0.57 | 0.22 | < 0.05 | 0.29 | 0.66 |
|  | 21 | 6.4^AY^ | 8.4^AY^ | 4.6^B^ | 5.7^B^ |  |  |  |  |  |  |  |  |
|  | 180 | 8^AX^ | 10.4^AX^ | 9.2^A^ | 7.4^A^ |  |  |  |  |  |  |  |  |
| Non-mitocondrial respiration | -45 | 6.0 | 4.7 | 5.1 | 4.6 | 0.5 | <0.0001 | 0.84 | 0.19 | < 0.05 | 0.12 | 0.18 | 0.20 |
|  | 21 | 5.2 | 6.8 | 6.5 | 6.7 |  |  |  |  |  |  |  |  |
|  | 180 | 6.5 | 7.1 | 8.1 | 7.2 |  |  |  |  |  |  |  |  |
| Respiratory control ratio | -45 | 4.4^a^ | 4.5^a^ | 4.1^a^ | 4.0^a^ | 0.6 | < 0.01 | 0.099 | 0.9 | 0.099 | 0.86 | < 0.01 | 0.15 |
|  | 21 | 2.9^a^ | 5.1^a^ | 5.0^a^ | 3.5^a^ |  |  |  |  |  |  |  |  |
|  | 180 | 3.0^a^ | 3.1^b^ | 4.6^a^ | 2.4^b^ |  |  |  |  |  |  |  |  |
