# Supplementary material for: Hepatic metabolism of grazing cows of two Holstein strains under two feeding strategies with different levels of pasture inclusion
Source: PLoS One. 2023 Oct 26;18(10):e0290551. doi: 10.1371/journal.pone.0290551 (PMC10602316; doi:10.1371/journal.pone.0290551)
Supplement: S3 Table — Data are shown as least square means ± standard error. ABabMeans having different uppercase superscripts differ significantly within feeding strategy across time period (P < 0.05). Means having different lowercase superscripts differ significantly within strain across time period (P < 0.05), while XYxymeans denote tendencies (0.05 < P < 0.1). N = 10–12. DIM: Days in milk; FS: Feeding strategy. 1Oxygen consumption rate measurements of liver biopsies were obtained after addition of 20 mM succinate, 4 μM ADP, 2 μM oligomycin, up to 4 μM carbonyl cyanide-p- trifluoromethoxyphenylhydrazone and 0.5 μM rotenone, and 2.5 μM antimycin. Oxygen consumption rates are expressed as pmol of O2 per min per mg of wet weight. (DOCX) [file pone.0290551.s004.docx]

| *Complex-II respiratory parameters^1^* | DIM | Treatments | | | | SEM | P-value | | | | | | |
| --- | --- | --- | --- | --- | --- | --- | --- | --- | --- | --- | --- | --- | --- |
|  |  | FixP | | MaxP | |  | DIM | Strain | FS | DIM x Strain | DIM x FS | Strain x FS | DIM x FS x Strain |
|  |  | NZH | NAH | NZH | NAH |  |  |  |  |  |  |  |  |
| State 3 respiration | -45 | 31 | 30 | 28 | 20 | 5 | <0.0001 | < 0.05 | 0.07 | 0.55 | 0.46 | < 0.001 | 0.41 |
|  | 21 | 26 | 33 | 36 | 24 |  |  |  |  |  |  |  |  |
|  | 180 | 49 | 56 | 58 | 37 |  |  |  |  |  |  |  |  |
| State 4 respiration | -45 | 18 | 15 | 15 | 12 | 3 | <0.0001 | 0.05 | 0.16 | 0.99 | 0.99 | < 0.05 | 0.06 |
|  | 21 | 19 | 21 | 23 | 14 |  |  |  |  |  |  |  |  |
|  | 180 | 31 | 36 | 39 | 25 |  |  |  |  |  |  |  |  |
| Maximum respiratory capacity | -45 | 46 | 46 | 39 | 28 | 9 | <0.0001 | 0.15 | 0.2 | 0.3 | 0.28 | < 0.01 | 0.41 |
|  | 21 | 29 | 42 | 47 | 31 |  |  |  |  |  |  |  |  |
|  | 180 | 66 | 75 | 79 | 46 |  |  |  |  |  |  |  |  |
| Oligomycin-resistant respiration | -45 | 17 | 15 | 16 | 13 | 3 | <0.0001 | < 0.01 | 0.21 | 0.31 | 0.40 | < 0.01 | 0.06 |
|  | 21 | 18 | 21 | 24 | 16 |  |  |  |  |  |  |  |  |
|  | 180 | 35 | 38 | 44 | 21 |  |  |  |  |  |  |  |  |
| Oligomycin-sensitive respiration | -45 | 14 | 15 | 11 | 8 | 2 | 0.13 | 0.54 | 0.08 | 0.62 | 0.46 | 0.01 | 0.84 |
|  | 21 | 8 | 12 | 13 | 9 |  |  |  |  |  |  |  |  |
|  | 180 | 15 | 18 | 13 | 9 |  |  |  |  |  |  |  |  |
| Non-mitocondrial respiration | -45 | 5.2 | 4.3 | 5.7 | 4.6 | 0.9 | <0.0001 | 0.76 | 0.15 | 0.19 | 0.89 | 0.76 | 0.82 |
|  | 21 | 5.6 | 5.5 | 5.4 | 5.9 |  |  |  |  |  |  |  |  |
|  | 180 | 8.7 | 8.8 | 9.1 | 10.1 |  |  |  |  |  |  |  |  |
| Respiratory control ratio | -45 | 1.7^A^ | 2.0^A^ | 2.0^A^ | 1.6^A^ | 0.1 | <0.0001 | 0.51 | 0.26 | 0.08 | < 0.01 | < 0.01 | 0.21 |
|  | 21 | 1.5^ABY^ | 1.6^ABY^ | 1.7^AX^ | 1.8^AX^ |  |  |  |  |  |  |  |  |
|  | 180 | 1.6^A^ | 1.5^A^ | 1.5^B^ | 1.2^B^ |  |  |  |  |  |  |  |  |
