# Supplementary material for: Hepatic metabolism of grazing cows of two Holstein strains under two feeding strategies with different levels of pasture inclusion
Source: PLoS One. 2023 Oct 26;18(10):e0290551. doi: 10.1371/journal.pone.0290551 (PMC10602316; doi:10.1371/journal.pone.0290551)
Supplement: S4 Table — Data are shown as least square means ± standard error. N = 10–12. DIM: Days in milk; FS: Feeding strategy. (DOCX) [file pone.0290551.s005.docx]

|  | DIM | Treatments | | | | SEM | P-value | | | | | | |
| --- | --- | --- | --- | --- | --- | --- | --- | --- | --- | --- | --- | --- | --- |
|  |  | FixP | | MaxP | |  | DIM | Strain | FS | DIM x Strain | DIM x FS | Strain x FS | DIM x FS x Strain |
|  |  | NZH | NAH | NZH | NAH |  |  |  |  |  |  |  |  |
| CPT specific activity (mU.mg^-1^) | -45 | 4.89 | 4.91 | 3.82 | 4.36 | 0.47 | <0.0001 | 0.10 | < 0.05 | 0.16 | 0.63 | 0.45 | 0.92 |
|  | 21 | 2.00 | 3.31 | 1.39 | 2.84 |  |  |  |  |  |  |  |  |
|  | 100 | 4.51 | 5.57 | 2.87 | 4.75 |  |  |  |  |  |  |  |  |
|  | 180 | 4.73 | 4.17 | 4.86 | 3.59 |  |  |  |  |  |  |  |  |
