# Supplementary material for: Hepatic metabolism of grazing cows of two Holstein strains under two feeding strategies with different levels of pasture inclusion
Source: PLoS One. 2023 Oct 26;18(10):e0290551. doi: 10.1371/journal.pone.0290551 (PMC10602316; doi:10.1371/journal.pone.0290551)
Supplement: Attachment — Submitted filename: Reviews.docx [file pone.0290551.s006.docx]

Journal Requirements:

When submitting your revision, we need you to address these additional requirements.

1. Please ensure that your manuscript meets PLOS ONE's style requirements, including those for file naming. The PLOS ONE style templates can be found at 
<https://journals.plos.org/plosone/s/file?id=wjVg/PLOSOne_formatting_sample_main_body.pdf> and 
<https://journals.plos.org/plosone/s/file?id=ba62/PLOSOne_formatting_sample_title_authors_affiliations.pdf>

2. Thank you for stating the following financial disclosure: 
"Funding
M. García-Roche was supported by CAP fellowship BDDX_2018_1#49004502. D. Talmón was supported by ANII fellowship POS_NAC_2017_1_141266.  A. Cassina and C. Quijano were partially funded by grants of the Espacio Interdisciplinario – Centros, UDELAR 2015. A. Cassina was also supported by the grant CSIC grupos I+D 2014 (767). The project was funded by Comisión Sectorial de Investigación Científica (CSIC) of the Universidad de la República (UdelaR) CSIC I+D 2018 ID 103 to M. Carriquiry and C. Quijano as well as by Agencia Nacional de Investigación e Innovación (ANII) INNOVAGRO 2018: FSA_1_2018_1_152220 to M. Carriquiry and A. Cassina. A. Mendoza received funding from the project PL_21_0_00 of INIA."

Please state what role the funders took in the study.  If the funders had no role, please state: "The funders had no role in study design, data collection and analysis, decision to publish, or preparation of the manuscript." 
If this statement is not correct you must amend it as needed. 
Please include this amended Role of Funder statement in your cover letter; we will change the online submission form on your behalf.

AU: Sentence added.

3. Please expand the acronym “CAP, ANII, and INIA” (as indicated in your financial disclosure) so that it states the name of your funders in full.
This information should be included in your cover letter; we will change the online submission form on your behalf.

AU: Thank you, acronyms were expanded.

4. We note that the grant information you provided in the ‘Funding Information’ and ‘Financial Disclosure’ sections do not match.

When you resubmit, please ensure that you provide the correct grant numbers for the awards you received for your study in the ‘Funding Information’ section.

5. PLOS ONE now requires that authors provide the original uncropped and unadjusted images underlying all blot or gel results reported in a submission’s figures or Supporting Information files. This policy and the journal’s other requirements for blot/gel reporting and figure preparation are described in detail at <https://journals.plos.org/plosone/s/figures#loc-blot-and-gel-reporting-requirements> and <https://journals.plos.org/plosone/s/figures#loc-preparing-figures-from-image-files>. When you submit your revised manuscript, please ensure that your figures adhere fully to these guidelines and provide the original underlying images for all blot or gel data reported in your submission. See the following link for instructions on providing the original image data: <https://journals.plos.org/plosone/s/figures#loc-original-images-for-blots-and-gels>. 

In your cover letter, please note whether your blot/gel image data are in Supporting Information or posted at a public data repository, provide the repository URL if relevant, and provide specific details as to which raw blot/gel images, if any, are not available. Email us at [plosone@plos.org](mailto:plosone@plos.org) if you have any questions.

AU: Added to supporting information (S5 file).

6. Please clarify the Table 6 "Table 6. Inhibition by diphenyliodonium in liver biopsies of North American (NAH) and New Zealand (NZH) grazing Holstein cows supplemented with total mixed ration (FixP) or an energy-protein concentrate (MaxP) during prepartum and early lactation." in page "25" and Table 6 "
Table 6. Hepatic gene expression of North American (NAH) and New Zealand (NZH) grazing Holstein cows supplemented with total mixed ration (FixP) or an energy-protein concentrate (MaxP) at 180 DIM." in page "29".

AU: Numbering has changed.

7. Please include captions for your Supporting Information files at the end of your manuscript, and update any in-text citations to match accordingly. Please see our Supporting Information guidelines for more information: <http://journals.plos.org/plosone/s/supporting-information>.

 AU: Captions included in the manuscript.

Additional Editor Comments:

Dear Authors,
We have now received the required feedbacks from two reviewers and, before recommending your work for publishing, I think you should address the aspects they have highlighted, to further improve the quality of your manuscript. Thanks for your contribution to the Plos One. Best regards, Arda Yıldırım


[Note: HTML markup is below. Please do not edit.]

Reviewers' comments:

Reviewer's Responses to Questions

**Comments to the Author**

1. Is the manuscript technically sound, and do the data support the conclusions?

The manuscript must describe a technically sound piece of scientific research with data that supports the conclusions. Experiments must have been conducted rigorously, with appropriate controls, replication, and sample sizes. The conclusions must be drawn appropriately based on the data presented.

Reviewer #1: Partly

Reviewer #2: Partly

2. Has the statistical analysis been performed appropriately and rigorously?

Reviewer #1: Yes

Reviewer #2: Yes

3. Have the authors made all data underlying the findings in their manuscript fully available?

The [PLOS Data policy](http://www.plosone.org/static/policies.action#sharing) requires authors to make all data underlying the findings described in their manuscript fully available without restriction, with rare exception (please refer to the Data Availability Statement in the manuscript PDF file). The data should be provided as part of the manuscript or its supporting information, or deposited to a public repository. For example, in addition to summary statistics, the data points behind means, medians and variance measures should be available. If there are restrictions on publicly sharing data—e.g. participant privacy or use of data from a third party—those must be specified.

Reviewer #1: Yes

Reviewer #2: Yes

4. Is the manuscript presented in an intelligible fashion and written in standard English?

PLOS ONE does not copyedit accepted manuscripts, so the language in submitted articles must be clear, correct, and unambiguous. Any typographical or grammatical errors should be corrected at revision, so please note any specific errors here.

Reviewer #1: Yes

Reviewer #2: Yes

5. Review Comments to the Author

Please use the space provided to explain your answers to the questions above. You may also include additional comments for the author, including concerns about dual publication, research ethics, or publication ethics. (Please upload your review as an attachment if it exceeds 20,000 characters)

Thank you for your meaningful and constructive reviews. We have included more information and references to a publication of the same animal trial where animal and whole-farm biophysical performance were studied to support the Experimental Design section in Materials and Methods. In an effort to make the Results section more straight-forward, we avoided results which could translate into redundancies. For this, we excluded the free liver glucose to glycogen and liver glycogen to triglyceride ratios, non-significant correlations and the table that contained the strain x FS interaction for mitochondrial respiratory parameters as we found this information to be contained in other results. We also reanalyzed our results and modified the figures and tables to include all interactions. Figures were modified to include both two-way interactions: DIM x strain and DIM x FS and tables were modified to show only strain x FS means and all effects (fixed effects and two-way and three-way interactions)- Figures with the interaction strain x FS were included when found useful for the interpretation of results. In figure 5 we noticed that the graph with the correlation had incorrect x-axis legend, it was meant to be oligomycin-sensitive respiration, we included this correlation in the discussion section. Also, the order of the results was modified so it matched the order of the Discussion section. Finally, we modified the discussion by separating it into subsections with the headings: “Effect of strain on stage of lactation”, “Effect of feeding strategy on stage of lactation” and “Effect of feeding strategy and strain”.

Reviewer #1: This manuscript describes an experiment evaluating hepatic metabolism of 2 Holstein strains in 2 feeding strategies of a grazing dairy at multiple time points during lactation. Overall, the experiment was performed well and data presentation is clear, but discussion of results is poor. The effect of treatments (3-way or 2-way interactions and main effects) on variables of hepatic metabolism are not consistent making interpretation very difficult. The current Discussion section does not help the reader interpret results at all. The Discussion section needs to be rewritten focusing on interpretation of results as to evaluating the appropriate strain for a particular feeding strategy or vice versa.

Specific comments:
L27 - please indicate that repeated measures was involved in this analysis

AU: Thank you, it was added.

L30 - change to 'MaxP strategy at 21 DIM'

AU: Thank you, it was changed

L31 - change to 'MaxP strategy at 21 DIM'

AU: Thank you, it was changed

L56 - here and many other places in the manuscript you use a hyphen rather than a dash. The dash was correctly used in L55. please correct all occurrences.

AU: Thank you, checked.

L62 - change to 'associated with an increased'

AU: Thank you, it was changed

L73 - change to 'associated with increased'

AU: Thank you, it was changed

L105 - this is a 1 sentence paragraph

AU: Thank you, it was changed

L111 and 112 - 'presented' should be 'represented'

AU: Thank you, it was changed

L120 - is this referring to pasture intake, if so how did you measure that, or herbage allowance, if so how did you measure herbage mass

AU: Pre and post grazing pasture height was measured using a C-Dax Pasture Meter to estimate pasture intake, further detail was added to Materials and Methods. However, this information is present in a publication that specifically studied pasture growth and DM intake: - Stirling, S., L. Delaby, A. Mendoza, and S. Fariña. 2021. Intensification strategies for temperate hot-summer grazing dairy systems in South America: Effects of feeding strategy and cow genotype. J. Dairy Sci. 104 https://doi .org/10.3168/jds.2021-20507.


L123 - is 'paired' the block effect mentioned later in statistical analysis? If so you should use block term here

AU: Yes, it was changed for “blocked”.

L126 - how do you know that pasture DMI was 33% of total DMI? did you measure pasture DMI?

AU: Thank you, lines were added.


L131 - what time of day were cows milked and how were they milked?

AU: Milking time was added, cows were milked in a milking parlor with automated milk extraction, and automatic cluster removal. The latter information is not included since it is far from the objectives of this work, however the recording system was added.

L133-134 - this sentence is confusing. what is annual average referring to?

AU: More information was added in Materials and Methods to clarify,

L141 - how do you measure half a leaf

AU: It is in average two to three tillers or three tillers, however this information was excluded and a reference is provided to a publication that specifically studied pasture growth and DM intake : - Stirling, S., L. Delaby, A. Mendoza, and S. Fariña. 2021. Intensification strategies for temperate hot-summer grazing dairy systems in South America: Effects of feeding strategy and cow genotype. J. Dairy Sci. 104 https://doi .org/10.3168/jds.2021-20507.


Table 1 - diet composition at -45 DIM is missing but the chemical composition is shown in Table 2

AU: Prepartum diet was written in the text, it was now included to table 1.

Table 2 - it is somewhat difficult to discern which columns fall under which headings

AU: Thank you, lines were added.

L186 - I assume since liver biopsies were cryopreserved that all mitochondrial respiration measurements for different DIM were performed at the same time. Measurements at different times would be affected by the consistency of the respiration medium making comparison of different DIM less relevant.

AU: Thank you, we have clarified this observation in the Materials and Methods section. Lines 204-205

L230 - change to 'addition of the specific'

AU: Thank you, it was changed.

L240 - why were only 3 treatments measured for fatty acid driven respiration

AU: Thank you, we have clarified this observation in the Materials and Methods section. Lines 260-261

L264 - change to 'presented in units'

AU: Thank you, it was changed.

L290 - are these the same 8 cows used for Western blots? Can PCR and western blot results be compared?

AU: Thank you, we have clarified this observation in the Materials and Methods section. Lines 205-206

L314 - change to '(TNFA, and tumor'

AU: Thank you, it was changed.

L338 - is cow the correct experimental unit. I assume cows grazed as a group, but were they fed concentrate or TMR individually?

AU: Further detail is provided in the Materials and Methods section. The effect of strain is individual to every cow. With respect to the feeding strategies, the experiment consisted of a farmlet design that simulated two feedings strategies that reflect commercial practice (Stirling et al., 2021). In the MaxP strategy, cows were offered pasture depending on the pasture growth rate, with a heavy control on pasture growth. On the other hand, the FixP strategy had a moderate control on pasture growth and supplementation with TMR was offered on feedpads. To avoid dominance, cows grazed as a group within each strain and were all multiparous to avoid dominance. Also, cows were allocated to daily strips in a rotational manner to avoid any confounding effect from a given paddock. Concentrate was fed individually in the milking parlor to cows of the MaxP strategy, however, the TMR was offered in feedpads. In addition, the fact that most variables consisted of repeated measures minimizes the individual effect of the cow as previously mentioned by Bello et al., (2016).

L343 - how many variables had more than 3 outliers

AU: There were not variables with more than 3 outliers, variables with non-normal distribution were log-transformed. This observation was clarified in lines 360-362
Table 3 - the P-value for strain x FS is missing

AU: It was not included because it was not significant. It has now been included in the new table.

L394 - this is a 1 sentence paragraph

L399 - what is meant by moment of lactation? I am not familiar with that term and likely most readers are not either

AU: Thank you, it was changed to stage of lactation.

Table 4 - how were glycogen/glucose ration and triglyceride/glycogen ratio calculated. my calculations based on data in the table do not match the values reported

AU: Thank you for your observation. Both ratios have a considerably high SEM, we have decided to exclude these variables from our results section. They do not provide additional information and may lead to misinterpretation of results. Also, we have made an effort to reduce the results section for a better interpretation of results.

L444 - change to 'interaction of strain'

AU: Thank you, it was changed.

L455 - change to 'between DIM and FS' to use consistent terminology

AU: Thank you, it was changed.

L468 - 'Alongside' seems unusual term here. suggest changing to 'Also'

AU: Thank you, it was changed.

Table 6 - why only -45 and 21 DIM. And why was FS not evaluated in this table
AU: As we worked with cryopreserved samples and a great amount of samples we decided to prioritize this analysis for the transition period which is the period when dairy cows are most susceptible to metabolic disorders. Since no other interactions were significant we had not added this information, it has now been added. Lines 245-247.

L524 - need to define AcK

AU: Thank you, it was defined.
L590 - why was CD36 only measured in NAH cows

AU: All data on gene expression is provided in table 8, however graphs were performed only when P values were significant or tended to be significant to for better interpretation of results since over 20 genes were studied. Further detail is provided in the Materials and Methods section related to which results are presented in tables and which in graphs, lines 380-386.

L748 - change to 'content. North American'

AU: Thank you, it was changed.

L752 - change to 'metabolism in this strain.'

AU: Thank you, it was changed.

L748-756 - this explanation of the results is understandable and meaningful

AU: Thank you for your remark, we have modified the manuscript so it follows this rationale.

Figure 2B - The interaction was not significant but looks like the means separation is indicating an interaction is present

AU: Thank you, we agree with your observation, however it is the result we obtained from the statistical analysis. Bear in mind, the model includes the fixed effects of DIM, strain, FS and the corresponding two-way and three-way interactions. We have, nonetheless, excluded this figure in an effort to reduce the results section for a better interpretation of results.

Figure 3 - this seems to be the exact same data as in Table 5

AU: Thank you, Table 5 was excluded, these results are visible in Figure 4 and Tables 1 and 2 of Supporting Information S2.

Supplemental Table 1 and 2 - the superscripts seem to indicate there is a 3-way interaction but the p-value does not indicate an interaction. need to use different letters for superscripts to separate means of different main effects and interactions (abc, xyz, ABC, etc.)

AU: Thank you, it was changed

Supplemental Table 1 - what is DIMPP

AU: Thank you, it was changed

Reviewer #2: The manuscript characterized adaptations of hepatic metabolism of dairy cows of two Holstein strains with varying proportions of grazing in the feeding strategy.The design of the study is very interesting, the result confirmed the association between liver triglyceride, decreased hepatic mitochondrial function and greater mitochondrial acetylation levels in cows with a higher inclusion of pasture and suggests differential adaptative mechanisms between NAH and NZH cows to strategies with varying proportions of grazing in the feed strategy. The main problem is, liver metabolism changes need to be related to the difference of nutrient intake, digestive and metabolic rate.

Thank you for your meaningful and constructive reviews. We have included estimated nutrient intake in Materials and Methods and emphasized reduced dry matter intake in pasture-based systems in the Discussion section.

Some specific comments:
1.L113. Please give us more details of the “economic and productive selection index”.

AU: Thank you, more information on the national economic-productive breeding value was included and two references were added (124-127 lines).

2.L121. How did you measure the intake of mixed pasture? Were the intakes of NZH, NAH cows designed at the same intake/body weight^0.75 ratio? If not, why the intake of NZH, NAH cows were designed at different level ?

AU: Pre and post grazing pasture height was measured using a C-Dax Pasture Meter to estimate pasture intake, further detail was added to Materials and Methods (150-157 lines). Predicted DM intake was estimated with NRC 2001 which takes into account fat corrected milk, metabolic BW and week of lactation. This information is present in a publication that specifically studied pasture growth and DM intake: - Stirling, S., L. Delaby, A. Mendoza, and S. Fariña. 2021. Intensification strategies for temperate hot-summer grazing dairy systems in South America: Effects of feeding strategy and cow genotype. J. Dairy Sci. 104 https://doi .org/10.3168/jds.2021-20507.

3.Table 1. The total offered dry matter of NZH, NAH are different within the same stage. Intake level will affect the groth and metabolism of animals.

AU: Thank you for the remark, table 1 was incorrectly named, it is the estimated dry matter intake according to pre and post grazing pasture height and TMR and concentrate offered and refusals. Pasture allowance was allocated according to pasture growth rate and predicted DM intake. Further detail has been included in the Materials and Methods section for clarification.

We agree intake level may affect growth and metabolism of animals, as mentioned in the Introduction (66-71 lines) and Discussion (733-737 and 781-790 lines) intake is limited in pasture-based systems and becomes more limited with increasing proportions of grazed pasture.

4.Table 1. Please calculate the main nutrient (crude protein, NDF, ADF, ME,et al.) intake.

AU: Thank you, it was included in table 1.

5.Table 3. The a,b,c or x,y indicate that the data in the same row or line differ. Please specify whether the comparison is among the row or line. The superscripts are mislabeled in some rows, for example: milk yield at 21 DIM, superscript c did not appear, at 180 DIM, superscript a did not appear, and so on.Please check them carefully.

AU: Comparisons are not within the same row, they are within the same variable, letters c and d in milk yield do not appear in 21 DIM because milk yield is lower at 180 DIM, vice versa for letter a in 180 DIM.

However, to clarify the interpretation of results, we have included two panels: one for DIM x Strain interaction and another for DIM x FS interaction while the Strain x FS interaction is presented mostly in tables, except for two figures to aid with the discussion of results.

6.Fig.2. Please show us which one is NZH or NAH. The same as some other figures.

AU: To clarify the interpretation of results, the results section was modified in order to show DIM x FS in one panel and DIM x Strain in the other, the Strain x FS interaction is shown in tables. The DIM x Strain interaction was not shown in graphs since it was not significant in most variables. Given that the three-way interaction was not significant for most of the results (only %protein in milk) it was not graphed so as to make interpretation of results more simple.

6. PLOS authors have the option to publish the peer review history of their article ([what does this mean?](https://journals.plos.org/plosone/s/editorial-and-peer-review-process#loc-peer-review-history)). If published, this will include your full peer review and any attached files.


If you choose “no”, your identity will remain anonymous but your review may still be made public.


**Do you want your identity to be public for this peer review?** For information about this choice, including consent withdrawal, please see our [Privacy Policy](https://www.plos.org/privacy-policy).

Reviewer #1: No

Reviewer #2: No
